# Shape variation in modern human upper premolars

**DOI:** 10.1371/journal.pone.0301482

**Published:** 2024-04-09

**Authors:** Petra G. Šimková, Lisa Wurm, Cinzia Fornai, Viktoria A. Krenn, Gerhard W. Weber

**Affiliations:** 1 Department of Evolutionary Anthropology, University of Vienna, Vienna, Austria; 2 Human Evolution and Archaeological Sciences HEAS, University of Vienna, Vienna, Austria; 3 Medical Technology Cluster, Business Upper Austria–OÖ Wirtschaftsagentur GmbH, Linz, Austria; 4 Department of Research in Occlusion Medicine, Vienna School of Interdisciplinary Dentistry–VieSID, Klosterneuburg, Austria; 5 Center for Clinical Research, University Clinic of Dentistry Vienna, Medical University of Vienna, Vienna, Austria; 6 Institute of Evolutionary Medicine, University of Zurich, Zürich, Switzerland; 7 Fraunhofer Austria Research GmbH, Graz, Austria; 8 Core Facility for Micro-Computed Tomography, University of Vienna, Vienna, Austria; University of Sulaimani College of Dentistry, IRAQ

## Abstract

Morphological variation in modern human dentition is still an open field of study. The understanding of dental shape and metrics is relevant for the advancement of human biology and evolution and is thus of interest in the fields of dental anthropology, as well as human anatomy and medicine. Of concern is also the variation of the inner aspects of the crown which can be investigated using the tools and methods of virtual anthropology. In this study, we explored inter- and intra-population morphometric variation of modern humans’ upper third and fourth premolars (P3s and P4s, respectively) considering both the inner and outer aspects of the crown, and discrete traits. We worked by means of geometric morphometrics on 3D image data from a geographically balanced sample of human populations from five continents, to analyse the shape of the dentinal crown, and the crown outline in 78 P3s and 76 P4s from 85 individuals. For the study of dental traits, we referred to the Arizona State University Dental Anthropology System integrated with more recent classification systems. The 3D shape variation of upper premolar crowns varied between short and mesio-distally broad, and tall and mesio-distally narrow. The observed shape variation was independent from the geographical origin of the populations, and resulted in extensive overlap. We noted a high pairwise correlation (r1 = 0.83) between upper P3s and P4s. We did not find any significant geographic differences in the analysed non-metric traits. Our outcomes thus suggest that geographical provenance does not play a determinant role in the shaping of the dental crown, whose genesis is under strict genetic control.

## Introduction

Dental morphology has been investigated widely in order to study the variation within and between species based on general shape, metric and non-metric traits [1–14]. The crown of the premolars is usually composed of two main cusps (buccal and lingual) each continuing into a root cone [15]. These cones can form two separate roots or be fused into one root. Rarely, a three-rooted premolar with one lingual and two buccal roots can be observed [11,15]. By position within the dental row and function, maxillary premolars are intermediate between front and molar teeth. In this respect, third premolars possess some functional characteristics of a canine, while fourth premolars are more engaged in grinding functions comparable to molars [6].

3D morphological variation of upper premolar crowns among modern human populations is poorly investigated since most of the studies focused on the roots and root canals [16,17] also in clinical settings [18–20]. Previous investigations of the human maxillary premolar variation using traditional metric approaches [21] and landmark-based multivariate techniques [22], but on the outer enamel surface (OES), revealed morphological differences that help to distinguish between modern human populations. On the other hand, upper premolar morphology delivered varying results for the purposes of taxonomic classification in paleoanthropological studies using non-metric trait analyses [2,23,24], and geometric morphometric approach delivered significant results only for the P4s [23]. Metric measurements such as bucco-lingual and mesio-distal crown dimensions or crown height have also been used to study premolar size variation between and within populations [3,25,26]. Other studies focused on the occurrence and expressions of non-metric traits such as the maxillary premolar accessory ridges [11,27,28], the premolar mesial and distal accessory cusp [11,29,30], or the essential crests [30] among various modern human populations.

Compared to the OES, the examination of the enamel-dentine junction (EDJ) presents advantages because it represents the primary developmental structure of the crown and is consequently the precursor of the enamel cap’s morphology [31–40], naturally resulting in a strong correlation between these two surfaces [31,33,41,42]. Furthermore, the overlying enamel protects the EDJ from early deterioration processes such as abrasion, erosion or chipping. The EDJ thus represents an appropriate alternative to the OES since it also bears relevant taxonomic information [31,37,39,43,44]. Morphometric studies of the EDJ of both permanent and deciduous molars are well represented in the literature [31,33,44–46], while for maxillary premolars, extensive comparative studies are very rare [47], or only focused on the descriptive assessment of the EDJ and OES [48–51]. Currently, 3D geometric morphometric (GM) analyses of the inner tooth crown variation in geographically diverse modern human samples have been published for permanent and deciduous molars [23,33,44,46,47,52], as well as lower premolars [53,54], and central incisors [55]. These studies did not detect geographically-dependent dental shape variation [33,54], thus, in the current study, we applied quantitative approaches to explore inter- and intra-population shape variation and covariation of modern human P3s and P4s, expecting to find similar outcomes. We want to test the hypothesis that the ranges of shape variation of upper premolars in different world populations overlap, meaning that recent human populations cannot be distinguished based on the shape of their upper premolar’s dentinal crown. We used high-resolution 3D image data and GMM to investigate a geographically heterogeneous sample consisting of modern human populations from Africa, South America, Europe, the Near East, Southeast Asia and Oceania. Size variation was investigated based on the natural logarithm of Centroid Size. Moreover, we analysed the expression of the non-metric trait on both the EDJ and the OES. Comprehensive knowledge of these factors is essential in biology for taxonomic and evolutionary research, including human genotype-phenotype associations, and is also relevant in the various fields of medicine including forensics and dentistry.

## Materials

We included 78 maxillary P3s and 76 maxillary P4s from 85 recent modern humans (Table 1). In particular, our sample comprised individuals from Oceania (n = 15), including the indigenous people of Papua New Guinea (n = 6), the former diverging from Eurasian populations around 62 to 75 thousand years ago [56–58], South America (n = 19), including the native population of Selk’nam from Tierra del Fuego (n = 7), Europe (n = 17), including Avars from the 8^th^ century (n = 7), sub-Saharan Africa (n = 15), Southeast Asia (n = 6) and the Near East (n = 14) including Bedouins (n = 4) and Natufians (n = 10). All teeth used for the EDJ analyses were free of decay or fillings and did not exceed Molnar’s [59] wear stage 3 (slight to moderate wear). Accordingly, populations from various geographical regions, climatic zones, and environments, featuring different life styles, were represented in our sample.

**Table 1:** List of third and fourth premolars (P3 and P4, respectively) used in this study. (Wear recorded according to Molnar, 1977; f = female; m = male; + = present;— = absent; N.A. = not available; EDJ = enamel-dentine junction).

*Population sample*	*Individual*	*Origin*	*Wear*		*Age (years)*	*Sex*	*P3 analyses*		*P4 analyses*	
			*P3*	*P4*			*EDJ*	*Outlines*	*EDJ*	*Outlines*
*Africans* ^a^	S16	Sub-Saharan	2	1	9–13	N.A.	+	+	+	+
	S29	Sub-Saharan	3	2	30–40	m	+	+	+	+
	S46	Sub-Saharan	2	2	14–18	f	+	+	+	+
	S61	Sub-Saharan	1	2	25–30	m	+	+	+	+
	S68	Sub-Saharan	2	2	25–30	m	+	+	+	+
	S97	Sub-Saharan	2	2	12 15	f	+	+	+	+
	S103	Sub-Saharan	-	2	12 15	m	-	-	+	+
	S111	Sub-Saharan	2	2	20–30	m	+	+	+	+
	S121	Sub-Saharan	2	2	25–30	m	+	+	+	+
	C333	Sub-Saharan	1	1	adult	N.A.	+	+	-	+
	ID_122_421_1464	Sub-Saharan	2	2	adult	m	+	+	+	+
	S85	Sub-Saharan	3	2	adult	N.A.	+	+	+	+
	S87	Sub-Saharan	2	2	adult	N.A.	+	+	+	+
	S128	Sub-Saharan	3	2	adult	N.A.	+	+	+	+
	S138	Sub-Saharan	2	2	adult	N.A.	+	+	+	+
*Europeans* ^a^^,^ ^b^	Cs428	Avar	3	2	16–18	m	+	+	+	+
	Cs495	Avar	1	1	7–8	N.A.	+	+	+	+
	Cs498	Avar	2	2	25–30	f	+	+	+	+
	Cs502	Avar	2	1	13–15	N.A.	+	+	+	+
	Cs541	Avar	3	2	19–30	f	+	+	+	+
	Cs582	Avar	3	2	19–25	f	+	+	-	+
	Cs654	Avar	1	1	3–5	N.A.	+	+	+	+
	ID_120_123_1043	Central European	1	1	10	m	+	+	+	+
	ID_300_510_578	Central European	1	1	11	f	+	+	-	-
	ID_125_028_1089	Central European	1	-	10	f	+	+	-	-
	ID_125_415_1124	Central European	-	1	6	m	-	-	+	+
	ID_120_074_711	Central European	1	1	6	m	+	+	-	+
	ID_122_510_1554	Central European	2	1	22	m	+	+	+	+
	ID_122_511_1555	Central European	2	2	adult	f	+	+	+	+
	Anatomy_19710	Central European	2	2	20	N.A.	+	+	+	+
	ID_126_804_1171	Central European	2	2	29	m	+	+	+	+
	ID_122_199_961	Central European	2	2	20	m	+	+	+	+
*Near Easterners* ^c^	BLZ_483	Bedouin	2	2	N.A.	N.A.	+	+	+	+
	BLZ_506	Bedouin	1	1	N.A.	N.A.	+	+	+	+
	BLZ_515	Bedouin	3	2	N.A.	N.A.	+	+	+	+
	NN1	Bedouin	3	3	N.A.	N.A.	+	+	+	+
	RCEH038	Bedouin	3	-	N.A.	N.A.	+	+	-	-
	AM_23	Natufian	2	2	N.A.	N.A.	+	+	+	+
	AM_56	Natufian	1	1	N.A.	N.A.	+	+	+	+
	AM_67	Natufian	3	2	N.A.	N.A.	+	+	+	+
	AM_69	Natufian	3	3	N.A.	N.A.	+	+	+	+
	AM_101	Natufian	3	2	N.A.	N.A.	+	+	+	+
	Hay_8	Natufian	4	4	N.A.	N.A.	-	+	-	+
	Hay_12	Natufian	1	1	N.A.	N.A.	+	+	+	+
	Hay_19	Natufian	2	2	N.A.	N.A.	+	+	+	+
	Hay_25	Natufian	3	3	N.A.	N.A.	+	+	+	+
*Oceanians* ^a^	C54	Oceanian	2	2	adult	m	+	+	+	+
	C55	Oceanian	3	3	adult	m	+	+	+	+
	C104	Oceanian	1	1	juvenile	N.A.	+	+	+	+
	S89	Oceanian	2	1	adult	N.A.	+	+	+	+
	NZ_1459	Oceanian	4	4	N.A.	N.A.	-	+	-	+
	NZ_3093	Oceanian	1	-	N.A.	N.A.	+	+	-	-
	NZ_3099	Oceanian	-	3	N.A.	N.A.	-	-	+	+
	NZ_3104	Oceanian	4	3	N.A.	N.A.	-	+	+	+
	NZ_3108	Oceanian	1	-	N.A.	N.A.	+	+	-	-
	CN5	Papuan	-	1	adult	m	-	-	+	+
	CN220	Papuan	1	2	adult	m	+	+	+	+
	CN230	Papuan	2	2	adult	m	+	+	+	+
	CN232	Papuan	2	2	adult	m	+	+	+	+
	CN236	Papuan	3	2	mature	m	+	+	+	+
	CN264	Papuan	2	1	30	m	+	+	+	+
*South Americans* ^b^	793	American	2	3	adult	m	+	+	+	+
	806	American	-	2	adult	m	-	-	+	+
	1169	American	1	1	juvenile	N.A.	-	+	+	+
	2286	American	2	2	N.A.	f	+	+	+	+
	3537	American	2	2	adult	N.A.	+	+	+	+
	5382	American	1	-	juvenile	N.A.	+	+	-	-
	5385	American	2	2	adult	N.A.	+	+	+	+
	5389	American	1	1	infant	N.A.	+	+	+	+
	5443	American	-	1	juvenile	f	-	-	+	+
	6321	American	3	3	adult	N.A.	+	+	+	+
	15353	American	1	2	adult	m	+	+	+	+
	TF_6031	Selk’nam	4	4	adult	N.A.	-	+	-	+
	TF_6034	Selk’nam	4	3	adult	f	-	+	+	+
	TF_6035	Selk’nam	3	3	adult	N.A.	+	+	+	+
	TF_6038	Selk’nam	3	-	adult	m	+	+	-	-
	TF_6040	Selk’nam	3	-	adult	f	+	+	-	-
	TF_6041	Selk’nam	4	3	adult	m	-	+	+	+
	TF_21462	Selk’nam	4	-	N.A.	N.A.	-	+	-	-
*Southeast Asians* ^b^	ID_122_342_1383	Indonesian	2	1	28	m	+	+	+	+
	ID_122_335_1376	Indonesian	3	3	36	m	+	+	+	+
	ID_122_369_1412	Indonesian	3	2	adult	m	+	+	+	+
	1365	Indonesian	4	-	N.A.	f	-	+	-	-
	1368	Indonesian	-	4	N.A.	f	-	-	-	+
	2583	South Chinese	4	4	N.A.	f	-	+	-	+

^a^University of Vienna, Department of Evolutionary Anthropology

^b^Natural History Museum, Vienna

^c^Tel Aviv University, Department of Anatomy and Anthropology, The Sackler Maculty of Medicine.

## Methods

### Scanning & segmentation

All specimens were scanned at the Vienna μCT Lab, Austria, with an industrial Viscom X8060 NDT scanner (scanning parameters: 110–140 kV, 280–410 mA, 1400–2000 ms, 0.75 mm copper filter with a voxel size ranging between 20 and 50 μm). Virtual segmentation of the μCT data was performed in Amira software (www.thermofisher.com) to obtain triangulated surface models of the enamel and dentine. Models were extracted from the left premolars but, if absent or unusable, the right-side premolars were considered (n = 49) since directional asymmetry is not expected in teeth [60]. If this was the case, the right-side datasets were flipped before surface extraction. Slightly worn dentinal horn tips were virtually reconstructed in Amira software by extending the contours of the existing part of the dentinal horn tips (n = 38).

### Reorientation of the models and data collection on the cervical and crown outlines

The segmented dental surface models were reoriented in Geomagic Design X 64 (www.3dsystems.com) according to an established protocol [61]. Thus, the best-fit plane of the cervical margin, or cervical plane, was computed and was used to reorient the dataset to set the cervical plane parallel to the x-y plane of the virtual working space. Afterwards, the crown was rotated to align the mesial ridge to the y-axis. Cervical and crown outlines of the reoriented surfaces were collected and projected onto the cervical plane. The outlines were imported into Rhinoceros 6 (www.rhino3d.com) and split into 24 curve segments by equiangular radial vectors that originated from the centroid of the outline. The intersection points between the radii and the outline marked 24 pseudo-landmarks.

### Landmark collection on the EDJ

The reoriented surfaces of the dentinal crowns were imported into the free-to-download EVAN-Toolbox software (www.evan-society.org) for further landmark collection. A total of 24 landmarks were placed on the EDJ of each of the premolars. On all premolars, two fixed landmarks were placed on the paracone and protocone horn tips. On the P3s, the remaining two fixed landmarks were placed at the deepest mesial and distal points of the central groove. Since the central groove is not as well defined in P4s, the landmarks were instead placed on the deepest points of the distal and mesial ridges, which are always well visible. Additionally, the marginal ridge of both tooth types was represented by 20 curve semilandmarks. The sliding of the semilandmarks was performed using the bending energy technique [62–64]. Our approach matched that performed on lower premolars in Krenn et al. [54], who validated this technique by assessing the observer error. We do not expect the validity of these protocols to change in the upper premolars.

### Geometric morphometric analyses

We performed GM analyses using the EVAN-Toolbox for each set of landmarks (namely, cervical outline, crown outline, EDJ, and dentinal crown) for P3s and P4s, separately. The dentinal crown dataset was created by merging the cervical outline and the EDJ landmark configurations. A General Procrustes Analysis (GPA [65]) was performed for all data sets to normalize the landmark configurations. The resulting Procrustes shape coordinates were analyzed via principal component analysis (PCA). The Thin-Plate Spline technique was used to visualize the shape changes along the principal components (PCs) by means of warping [62,66]. To explore the shape covariation between P3s and P4s, as well as between different aspects within the same dental type (i.e. between the cervical outline and the EDJ), we performed a 2-block partial least squares (2B-PLS) analysis. To analyze the effect of allometry, multivariate regression was performed. The size was investigated by using the natural logarithm of Centroid Size (lnCS [67]). By using the lnCS derived from the dentinal crown dataset (combining the EDJ with cervical outline) we obtained a measure of size representing crown height as well. For testing tooth size differences between populations, we performed Kruskal-Wallis and Mann-Whitney-U tests (in PAST 4.03; www.softpedia.com), since the obtained data was not normally distributed. Permutational Multivariate Analysis of Variance (PERMANOVA) was performed using PAST 4.03 to test for shape differences between the groups (using all PC-scores), allowing for a distribution-free setting using permutational algorithms.

### Non-metric traits

The following non-metric traits were considered:

1. Premolar mesial and distal accessory cusps
  Accessory mesial or distal cusps originate from a bifurcation on the margin of the sagittal sulcus that is separating the buccal and lingual cusps, forming a free-standing cusp on the OES [11,13,68]. Sakai et al. [69] describe accessory cusps as indistinct on the EDJ, with a protrusion or swelling of the marginal ridge where the cusp could be. We used the following classifications to evaluate the presence of the accessory cusps: absent (0), mesial cusp (1), distal cusp (2), and mesial and distal cusp (3).
2. Maxillary premolar accessory ridges (MxPAR)
  Accessory ridges may or may not be present on the lingual lobe segment of the buccal cusp, mesially and distally from the essential crest of both P3s and P4s [11], and can be extended into the central groove. These features are prevalent in both the OES and the EDJ, and their distribution was found to be heterogeneous among populations [28,70]. To represent the expression of these features, we used the following classification: absent (0), mesial accessory ridge (1), distal accessory ridge (2), and mesial and distal accessory ridges (3).
3. Essential crest
  The essential crests of the central lobe of the paracone and protocone are usually separated by the sagittal sulcus and central groove. However, if this is not the case, i.e., both essential crests are connected, they create a transverse crest. Additionally, the separated essential crest of the buccal cusp can also be bifurcated. Several studies were able to detect these features on both EDJ and OES [48–51]. In this study, we classified every kind of essential crest expression separately, namely the separated buccal and lingual crests (0), transverse crest (1), and the bifurcated buccal essential crest (2).

**Fig 1:**
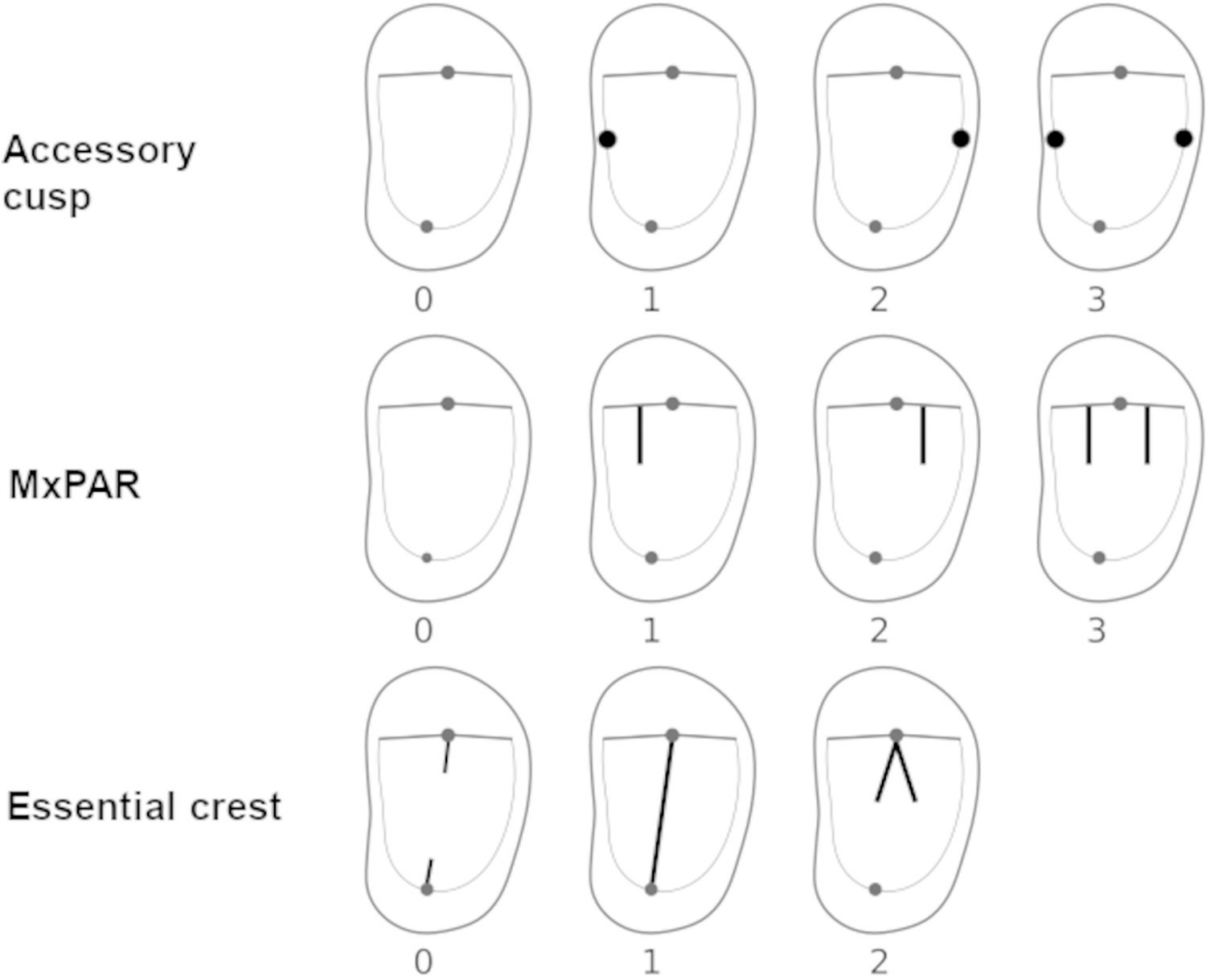
Illustration of scored non-metric traits and their expression categories.

Traits 1 and 2 were derived from the Arizona State University Dental Anthropological System [11,12]. For trait 3 (essential crest) we referred to Bailey’s [2] buccal essential crest and traverse crest grading system. All traits were scored both on the OES and EDJ of each specimen and evaluated according to their presence and expression (Fig 1), performing a Chí^2^ test in PAST 4.03.

### Ethics statement

In this study, we used non-invasive μCT (micro computed tomography) data (a three-dimensional X-ray method) from human remains included in the collections of the Department of Evolutionary Anthropology, University of Vienna and the Natural History Museum Vienna, Austria. Since only osteological material was used, no ethical approval or guidance was required as the research did not involve present-day human samples. The specimens were exported to Europe between the 19^th^ century and the beginning of the 20^th^ century, being curated in Austrian institutions for over a century. They were regarded and treated as precious parts of natural history collections and used for research since then. Today, their acquisition raises concerns according to current ethical principles. During the last 15 years, skulls were μCT-scanned at the Vienna μCT Lab of the University of Vienna for the purpose of preservation and fundamental research. Specimens were handled only by highly trained personnel in the most respectful way. No damages or alterations of any kind were caused to the remains.

## Results

### Shape variation in P3s

**Fig 2:**
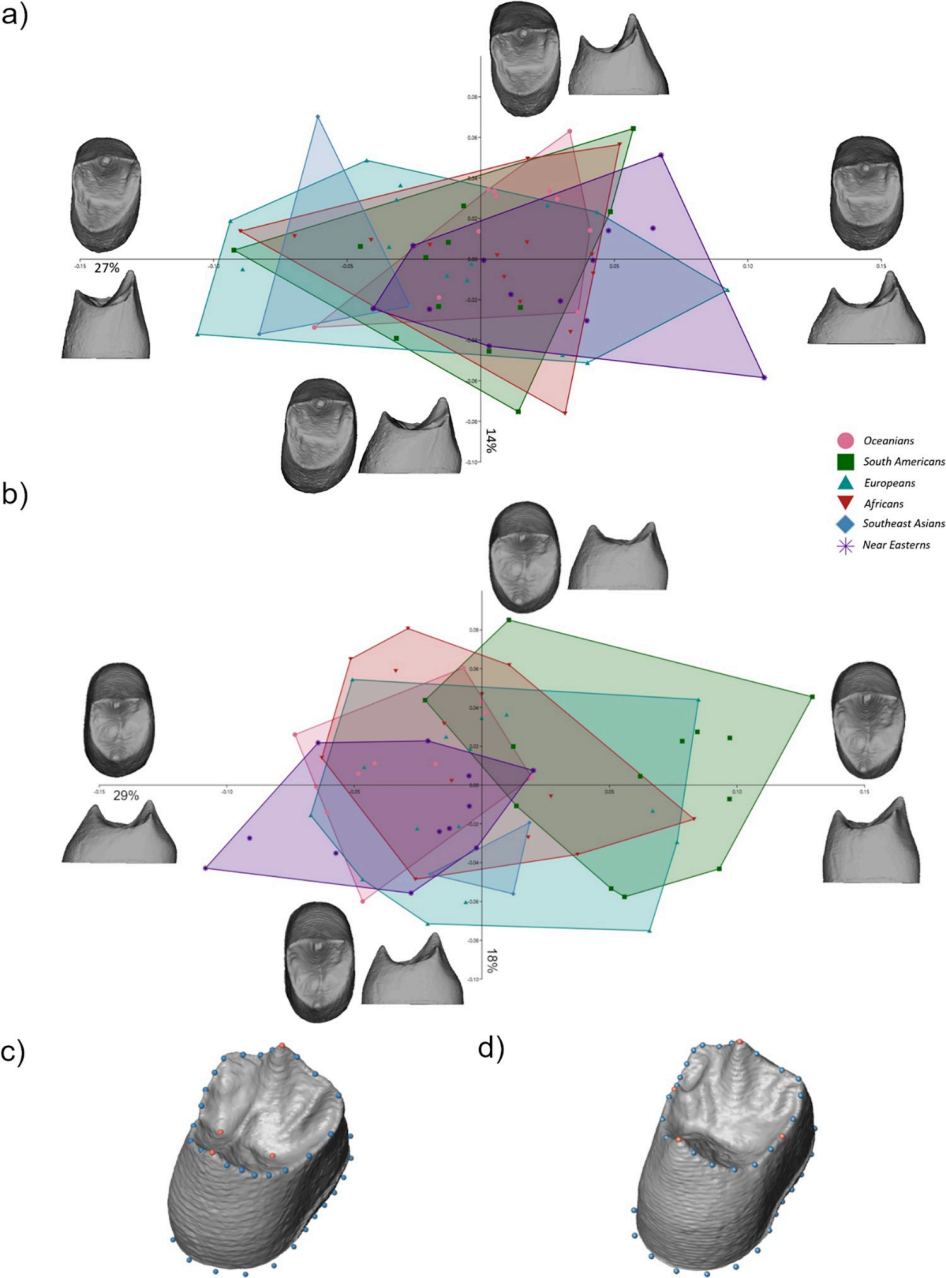
PC1—PC2 plot for third premolars (a) and fourth premolars (b) in shape space for the dentinal crown. The warpings show the real variation in occlusal and distal view at values PC1±0.15/ PC2 ±0.10; c) Landmark configuration capturing the dentinal crown of the P3s; and d) the P4s (orange = landmarks, blue = semi- and pseudolandmarks).

Results obtained from the PCA analysis of the P3 dentinal crown (Fig 2A) revealed an extensive overlap of the populations. Variation along PC1 (27% of the total variance explained) was mainly driven by the expansion of the distal fossa relative to the mesial fossa and the relative mesio-distal broadening of the cervical outline. The shape of the P3 dentinal crown varied from relatively short with a broad base, and rounded lingual occlusal aspect, to tall and mesiodistally narrow crowns. The horn tips shifted towards each other or apart, relative to the expansion and reduction of the cervical aspect of the crown, with broad crowns expressing horn tips closer to each other and narrow crowns the other way around. Shape variation along PC2 (14%) reflected the relative expansion of the mesio-buccal and mesio-lingual areas of the dentinal crowns with respect to each other. With the expansion of the mesio-lingual side, the relative position of the lingual cusp shifted mesially. Along PC3 (10%), the relative shape changes reflected the expansion of the distal aspect of the tooth as well as the relative position of the lingual cusp. The lingual cusp was more mesially positioned in disto-buccally expanded dentinal crowns, and disto-lingually positioned in disto-lingually expanded crowns.

### Shape variation in P4s

The shape of the P4 dentinal crowns (Fig 2B) along PC1 (29%) varied between short with mesio-distally expanded lingual cusp and bucco-lingually elongated crown base to tall with a mesio-distally narrow lingual cusp and bucco-lingually shorter crown base. A narrower occlusal aspect corresponds with a taller buccal cusp, positioned further from the lingual cusp relative to the crown base, while in broader occlusal aspects the cusps are more even and shifted towards each other. We observed a separation of Southeast Asian individuals from South Americans and Oceanians. However, this was probably caused by the small size of the Southeast Asian sample. On the other hand, Near Easterners and South Americans, although partially overlapping, tended to show the opposite shape variation along PC1. Shape variation along PC2 (18%) was mainly driven by the relative bucco-lingual position of the mesial fossa. A taller buccal cusp and relatively buccolingually elongated cervical outline were associated with a lingually shifted mesial fossa and vice versa. The variation along PC3 (14%) reflected the relative buccolingual position of the distal fossa as well as the height of the crown and relative expansion of the cervical outline.

The PCA analyses of the cervical outline, crown outline and EDJ dataset in both P3s and P4s (Supplementary Fig A, B, C, D in S1 File) showed that the populations in our sample overlapped widely and could not be differentiated based on the morphology of the upper premolars crown. Supplementary Table A in S1 File contains percentages of variance explained by the first five PCs in every dataset. Furthermore, our observations were confirmed by insignificant results obtained from the PERMANOVA analyses for P3s’ (*p* = 0.572), as well as P4s’ (*p* = 0.613) dentinal crowns, EDJ (P3, p = 0.53; p4, p = 0.87), cervical outlines (P3, p = 0.45; P4 = 0.06) and the P3s’ crown outlines (p = 0.17) (Supplementary Tables B and C in S1 File). We have found significant results only in the crown outline analysis for P4s (p = 0.03).

### Covariation

**Fig 3:**
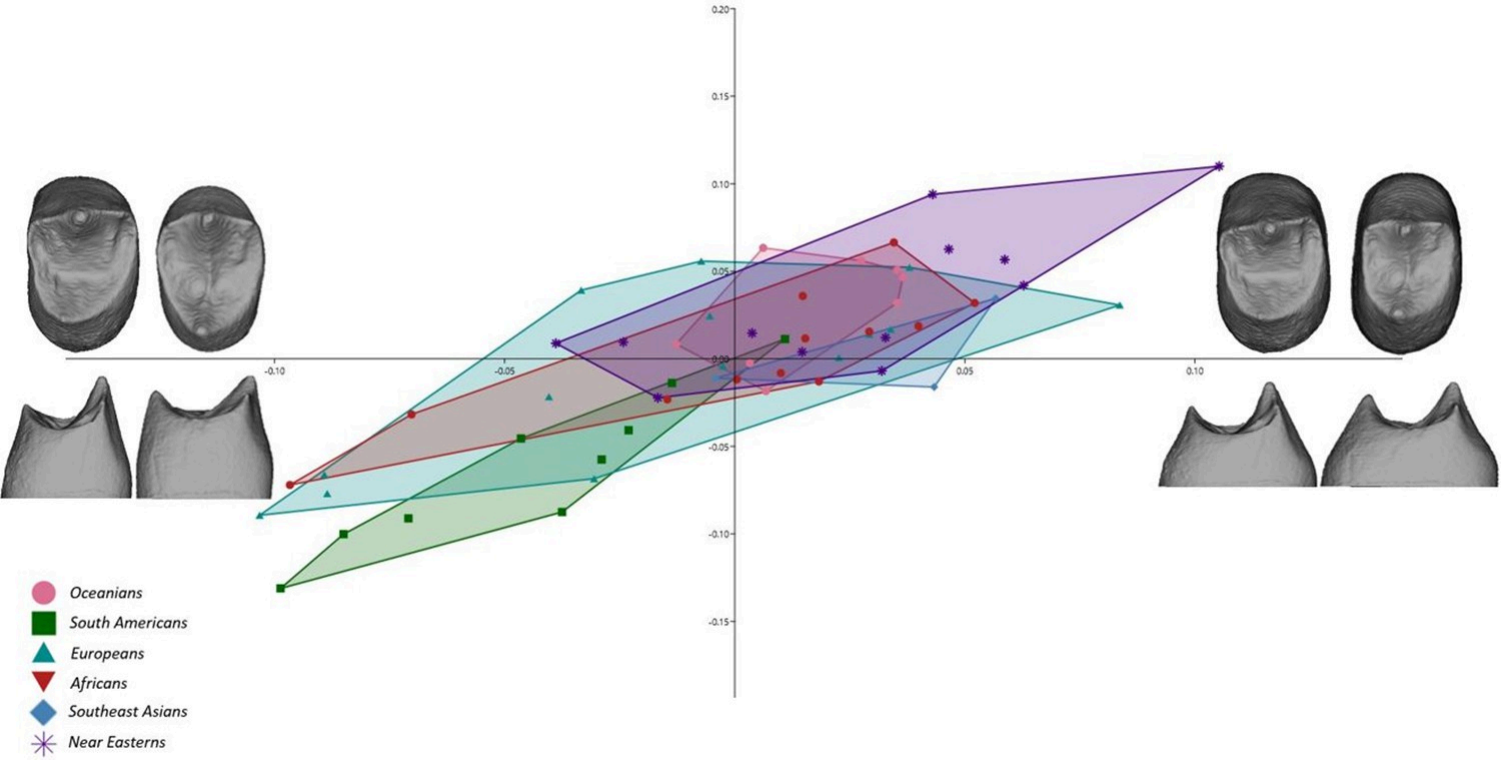
2B-PLS plot capturing the covariation of upper third and fourth premolar dentinal crowns (dataset combining enamel-dentine junction and cervical outline; the warping shows the real shape variation in occlusal and distal view at the extremities of the range of distribution).

The 2B-PLS analysis demonstrated a strong pairwise correlation (r1 = 0.83) between P3 and P4 dentinal crowns (Fig 3), which showed concurrent trends of dentinal crown shape variation. For the Singular Warp scores 1 of the dentinal crown analysis (explaining 81% of total covariance), both tooth types covaried between short-crowned with mesio-distally broad base or tall-crowned with mesio-distally narrow base. The occlusal aspect associated with the short and broad crown type was mesio-distally expanded and squared with a broad central groove. Tall- and slender-crowned teeth, on the other hand, showed mesio-distally reduced occlusal aspects and were lingually narrow. Furthermore, higher dentinal horn tips were observed in short-crowned P4s with respect to tall-crowned premolars.

The covariation between the EDJ occlusal aspect and the crown base (represented by the cervical outline) within tooth types was not as high as the covariation between the dentinal crowns of the two different tooth types. Nevertheless, pairwise correlation within the P4s parameters was higher (r1 = 0.56) than within P3s (r1 = 0.46) (Table 2).

**Table 2:** Results of the 2B-PLS (single warp score 1) for the upper third and fourth premolars (P3 and P4, respectively). (EDJ = enamel-dentine junction).

	Pairwise correlation (r1)	% of the total covariance
	P3 dentinal crown	P3 dentinal crown
P4 dentinal crown	0.83	81
	P3 cervical outline	P3 cervical outline
P3 EDJ	0.46	57
	P4 cervical outline	P4 cervical outline
P4 EDJ	0.56	70

### Size

**Fig 4:**
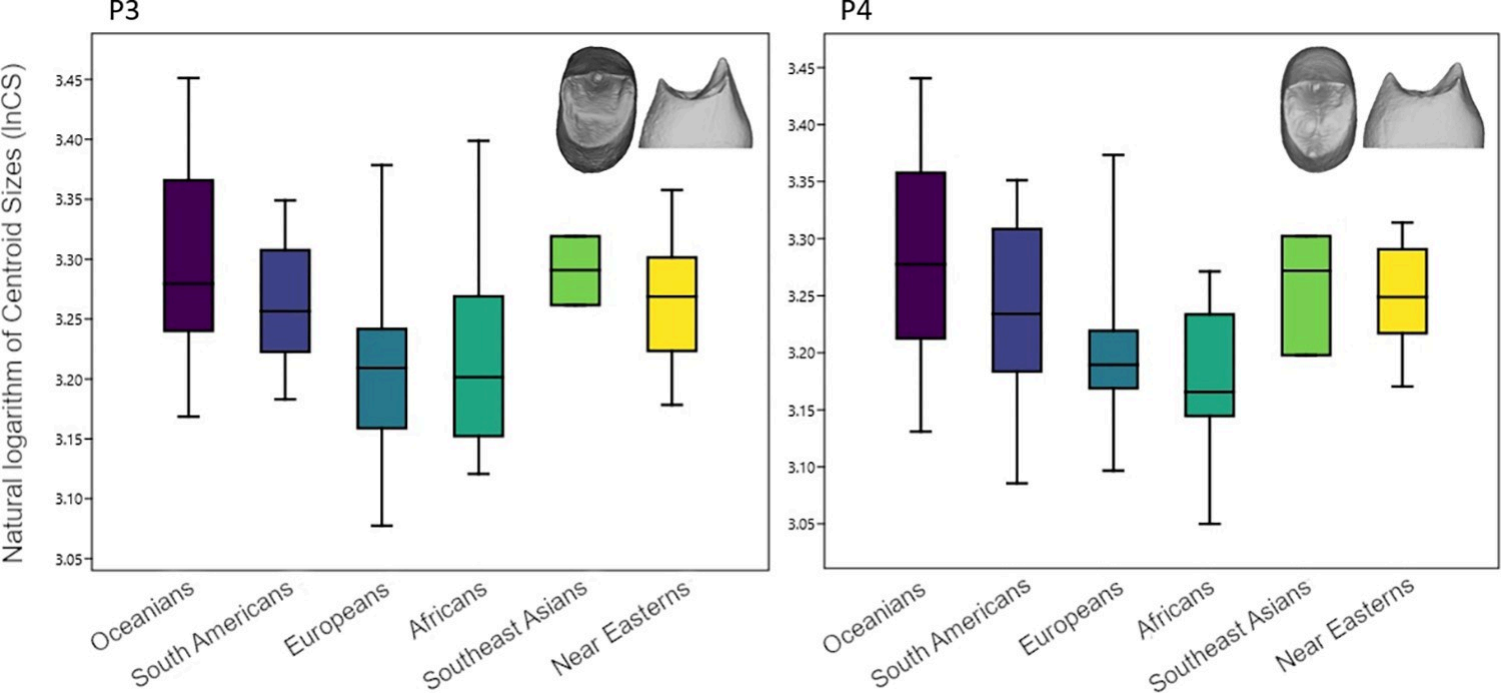
Boxplots of the natural logarithm of centroid sizes from the dentinal crown.

The lnCS was compared across the sample separately for the dentinal crown, cervical and crown outlines, and EDJ (Fig 4, Supplementary Fig E in S1 File). Kruskal-Wallis test showed significant differences for lnCS between all populations (Table 3). Comparing the groups against each other, the Mann-Whitney-U test showed that the Oceanian teeth were significantly larger than any other group for all features except the EDJ analysis. Similarly, Europeans and Africans were consistently smaller. Supplementary Table D in S1 File shows size differences between sexes in upper premolars, assessed via Mann-Whitney-U test.

**Table 3:** Results of the Kruskal–Wallis test. Differences in dental sizes (expressed by the logarithm of Centroid Sizes) for the upper third and fourth premolars (P3 and P4, respectively) among all populations across the sample. (EDJ = enamel-dentine junction).

	P3		P4	
	Kruskal-Wallis test	*p*-value	Kruskal-Wallis test	*p*-value
Cervical outline	21.20	**< 0.001**	19.51	**< 0.001**
Crown outline	25.54	**< 0.001**	14.06	**0.015**
EDJ	17.88	**0.003**	10.30	0.067
Dentinal crown	14.88	**0.01**	16.05	**0.006**

Multivariate regression of size on shape for dentinal crowns was performed to analyse allometry and showed that only 1.9% and 2.1% of the total P3, respectively P4 shape variance could be explained by size.

### Non-metric traits

Table 4 reports the prevalence of the premolar mesial and distal accessory cusps, accessory ridges and essential crest expressions on both the OES and the EDJ. Mesial and distal accessory cusps were present in only 10% of our sample. None of the Southeast Asian individuals exhibited this feature, and within the European, Near Eastern, South American and African samples, it could only be detected in a few individuals either on the OES or the EDJ of the P3 or P4. Various expressions of the accessory cusps on the OES were three times more frequent in P4s (12%) than in P3s (4%). Only five of these specimens expressed also an elevation of the marginal ridge on the EDJ. Interestingly, three accessory cusps were detected on the EDJ of the P3s and another three in the P4s, without any sign of expression on the OES.

**Table 4:** List of non-metric traits scored for the third and fourth premolars (P3 and P4, respectively). (Numbers 0,1,2 and 3 refer to the manifestation (see Methods); n = Number of individuals).

		Prevalence (%)							
P3		Trait expression	Oceania (n = 13)	South America (n = 16)	Europe (n = 16)	Africa (n = 14)	Southeast Asia (n = 3)	Near East (n = 13)	Complete sample
Accessory cusp	Enamel	0	85	100	100	100	100	93	96
		1	7	-	-	-	-	7	3
		2	8	-	-	-	-	-	1
		3	-	-	-	-	-	-	-
	Dentine	0	93	93	93	93	100	100	95
		1	7	7	7	-	-	-	4
		2	-	-	-	7	-	-	1
		3	-	-	-	-	-	-	-
Accessory ridges (MxPAR)	Enamel	0	69	50	44	36	67	39	48
		1	-	-	-	7	-	15	4
		2	8	31	25	43	33	15	25
		3	23	19	31	14	-	31	23
	Dentine	0	69	25	31	57	100	38	45
		1	-	6	6	7	-	8	5
		2	31	25	44	36	-	16	30
		3	-	44	19	-	-	38	20
Essential crest	Enamel	0	77	93	100	72	67	100	88
		1	15	7	-	14	-	-	7
		2	8	-	-	14	33	-	5
	Dentine	0	77	76	93	72	67	100	83
		1	15	12	7	14	-	-	9
		2	8	12	-	14	33	-	8
P4		Trait expression	Oceania (n = 13)	South America (n = 16)	Europe (n = 16)	Africa (n = 14)	Southeast Asia (n = 3)	Near East (n = 13)	Complete sample
Accessory cusp	Enamel	0	62	86	100	93	100	93	88
		1	31	-	-	7	-	-	7
		2	7	7	-	-	-	7	4
		3	-	7	-	-	-	-	1
	Dentine	0	84	93	100	73	100	100	91
		1	8	-	-	27	-	-	7
		2	8	-	-	-	-	-	1
		3	-	7	-	-	-	-	1
Accessory ridges (MxPAR)	Enamel	0	31	47	24	27	34	69	38
		1	8	-	6	13	33	8	8
		2	15	13	29	13	33	15	18
		3	46	40	41	47	-	8	36
	Dentine	0	86	13	30	40	67	54	35
		1	8	-	-	20	-	8	5
		2	8	7	23	7	33	15	14
		3	-	80	47	33	-	23	46
Essential crest	Enamel	0	100	100	93	74	67	92	91
		1	-	-	-	13	-	8	4
		2	-	-	7	13	33	-	5
	Dentine	0	93	100	88	74	100	84	88
		1	-	-	12	13	-	8	7
		2	7	-	-	13	-	8	5

MxPARs were present in 55% of P3s and 65% of P4s. In P3s, a distally located accessory ridge occurred commonly on both OES and EDJ. Conversely, most of the P4 specimens expressed accessory ridges in both mesial and distal positions. The highest frequency of expression of accessory ridges in P3s was found in South Americans (50% on the OES, 75% on the EDJ), while in P4s, Europeans possessed most MxPARs (76% on the OES, 70% on the EDJ).

Essential crest expression was homogenously distributed on both surfaces in both tooth types. Approximately 90% of the individuals possessed a simple essential crest, separated by the sagittal sulcus. The prevalence of the transverse crests and bifurcated crest expressions were comparable among the samples in both tooth types on both OES and EDJ.The results of the Chí^2^ test performed for every non-metric trait in both tooth types for both surfaces showed no significant difference in trait occurrence.

## Discussion

Dental development is generally under strong genetic influence resulting in constrained morphological variation of the dentition [71–73]. The applied 3D methodology delivered fine-scale results about the morphometric variation of premolars’ crowns. Maxillary premolars varied mainly in the length-to-breadth ratio of the occlusal aspect as well as the height of the dentinal crown and horn tips. A broader tooth shape is associated with short crowns but higher horn tips positioned closer to each other. On the contrary, narrow teeth displayed a tall crown but lower horn tips more apart from each other.

Based on our analyses of the dentinal crown, it was evident that the populations in our sample could not be characterized or distinguished by their upper premolars crown morphology, despite their different population history. A subtle divergence could be detected visually in the PCA plots for the P4 dentinal crown, separating Southeast Asians from Oceanians and South Americans, but a statistical shape difference between these groups could not be confirmed by the PERMANOVA. Nonetheless, it is interesting that similar observations were made for lower premolars [54]. However, this outcome should be confirmed using a larger sample since our results are based on 3 specimens only. Additionally, the overlap between South American and Near Eastern populations was minor. Prevailing short and broad posterior dental crown types including its extreme expressions, like in the Near Easterners, were already described for African populations [46,54]. As mentioned above, small sample sizes of various populations have to be acknowledged, as well as the different number of sub-samples within geographical populations. Our goal for this study was to create a sample containing as much diversity as possible, thus including more sub-samples from geographical regions. In this sense, our sample composition works against its harmonization, nevertheless we find that these world populations have very similar upper premolar shape variation. Since our research requires dental specimens with minimal signs of wear and abrasion, no dental treatments, and high-resolution scanning, living or recently deceased individuals are not usable for our study. While these are restrictions not in our favour, our approach allowed for an accurate 3D shape analyses of dentinal crowns, which are not accessible with traditional methods.

Compared to the dentinal crown and EDJ analyses, PERMANOVA analysis of the 2D outline datasets, revealed a couple of significant differences in group centroids in five out of sixty pairs of populations (Supplementary Table B in S1 File). Since the cervical and crown outline analyses do not contain 3D information, and thus are more similar to traditional linear measurements that have been proven nonreliable in population variation analyses [14], we suggest using them only in the absence of combined datasets rather than alone.

We interpret the strong covariation between P3 and P4 shapes (r1 = 0.83), both either broad and short, or narrow and tall, as a sign of a strong genetic component during tooth development in an environment with shared genetic influence [72,73]. This high correlation might be surprising, considering that P3s show a rather prominent buccal cusp and share the tearing function with the canines, while the P4s’ more even cusps engage in grinding activities, like in molars [8]. These are the first 3D outcomes on upper premolars and they are in agreement with those obtained for the lower premolars using a similar protocol [54], showing the same general pattern of variation. Thus, combining the evidence from our study and that of Krenn et al. [54], the dentinal crowns of both upper and lower P3s and P4s showed an extensive overlap between diverse populations that vary from short and broad to tall and narrow. European and Southeast Asian upper and lower premolars expressed extreme variations of the tall crown variant in both analyses. However, we found the relative cusp position of both cusps in upper P4s rather stable, with changes observable mainly in horn height, while in lower P4s, significant variation of the mesio-distal position of the lingual cusp was observed. The covariation expressed by upper and lower premolars can be explained by the need for morphological compatibility to guarantee stable occlusion and efficient function [6]. Since only the buccal cusp of the lower premolars is engaged at maximum intercuspation of the dentitions, the lingual cusp is less morphologically constrained [6,9,74]. Differently, in upper premolars both cusps are directly involved in occlusion [6], thus a lower degree of morphological variation might be expected. Consequently, the lingual aspect of the buccal cusp (paracone) and both the lingual and buccal aspects of the lingual cusp (protocone) should be more stable than other parts of the crown [3,6] because they are directly involved in occlusion.

Such constraints in shape variation could also be reflected in more local features. In our non-metric trait analysis, we observed a higher rate of trait expression on the paracone than on the protocone, in the central groove and on the marginal ridges, which would be in line with the predictions by Kraus et al. [6]. However, we also found considerable variation in the lingual facet of the paracone. Therefore, not all of our results support the assumption of higher morphological conservation due to functional constraints. On the other hand, non-metric traits are understood to be under strong genetic control and vary neutrally in comparison to metric traits [14,75], therefore we should not expect them to be strictly related to function only. Our findings were mostly consistent with those of the previous studies on non-metric traits focusing on the OES [2,3,13,21–23,27,28,68,76–78] as well as studies based on OES and EDJ [48–51,69]. However, in contrast to our results, Sakai et al. [69] reported that mesial and distal accessory cusps occurred more often in P3s than in P4s, working, however, only on Japanese dentitions. Overall, the rate of expression of accessory cusps in different human populations is heterogeneous, most of them observed in Oceanian individuals, while Southeast Asians and Europeans showed nearly none. Our findings are in agreement with Xing et al. [49], who found that accessory cusps on the OES are not necessarily associated with elevations on the EDJ. Turner et al. [68] also stated that there is no dentine involvement necessary in the formation of mesial and distal accessory cusps. Uneven enamel disposition can also correspond to accessory cusps, as well as other features displayed on the OES [38,79,80]. Similar patterns can be found in the derived forms of the essential crest that are more common in the African sample. We detected all different manifestations of the essential crest on the EDJ and OES of both tooth types; however, more often in P3s. Accessory ridges are also equally present on the EDJ and OES, where more than half of the P3s in our sample and nearly two-thirds of the P4s possessed an accessory ridge. This is consistent with the results of Burnett et al. [70] and Mihailidis et al. [28], which were, however, solely based on the OES. In agreement with these studies, accessory ridges in our sample were primarily present on the distal aspect of the tooth. In P3s, we observed the highest occurrence of accessory ridges in Europeans, followed by Africans and Near Easterners. In contrast to our work, Burnett et al. [70] found the highest frequency of accessory ridges in North-East Asian or Asian-derived populations and lower frequencies in Africans and Europeans. For the P4s, again, the distribution of accessory ridges in our sample was unequal, while Burnett et al. [70] described a relatively even distribution across the whole sample.

Regarding premolar crown size, Townsend’s [81] findings showed a significant sexual dimorphism for both maxillary premolars in the mesiodistal and buccolingual crown dimensions, with females possessing more broad/circular tooth crowns and males more narrow/ellipsoid crowns. We could not verify these results in our study due to the lack of reliable information about sex for most of our individuals. However, with regard to dental size, as represented by the lnCS, we did not find significant differences between males and females for the upper P4s, which parallels the results in Krenn et al. [54] for the lower premolars from a partially overlapping human sample. Conversely, we found significant differences for the upper P3s, but since our sample included more males (n = 26) than females (n = 9) our results should be interpreted with caution because of the uneven composition of the sample. According to Brace et al. [82] and Brace and Mahler [83], there is a gradient in increasing tooth size from north to south of the world. We indeed observed the smallest dental size in Europeans, while the largest teeth of our sample belonged to the Oceanian population. However, we also found African teeth of small size, which would not support Brace’s theory, but is in agreement with Hanihara and Ishida [24], who found sub-Saharan African dental sizes close to the average. Regardless of the unverifiable impact of sex on our results, we can state that static allometry in maxillary premolars is generally quite small in our sample. The effect of size was also small, explaining about 2% of the shape variation within our sample.

Our study generally demonstrated a generally large morphological variation of modern human upper premolars, yet a high correlation between first and second upper premolar. This variation does not seem to be the result of geographical separation in the course of modern human evolution, since most studied populations from different continents, climatic zones and environments did not differ from each other strong enough to be discriminated based on their upper premolar morphology. Our results instead show that the upper premolar morphological variation of recent modern humans is very similar among world populations, and thus, based on our data, we accept the tested hypothesis that the ranges of upper premolar shape variation of various human populations overlap and they cannot be distinguished based on their upper premolar’s dentinal crown morphology only. For evolutionary studies, comparing modern humans with archaic hominins or non-human primates, this means that the geographic composition of the comparative modern human sample is of lesser importance. More work is needed to unravel the mechanisms behind this concordance of premolar shape independent of geographical origin.

## Supporting information

S1 File Šimková et al. Supporting information.
(DOCX)
