# Supplementary material for: Shape variation in modern human upper premolars
Source: PLoS One. 2024 Apr 9;19(4):e0301482. doi: 10.1371/journal.pone.0301482 (PMC11003632; doi:10.1371/journal.pone.0301482)
Supplement: S1 File — Supporting information. (DOCX) [file pone.0301482.s001.docx]

| **Table A. Percentage of variance** explained by the first five PCs (principal components) as resulting from the analysis of the upper third and fourth premolars’ (P3 and P4, respectively) cervical outline, crown outline, the enamel-dentine junction (EDJ) and combined EDJ and cervical outline (dentinal crown). | | | | | | | | |
| --- | --- | --- | --- | --- | --- | --- | --- | --- |
|  | P3 | | | | P4 | | | |
|  | Cervical outline | Crown outline | EDJ | Dentinal crown | Cervical outline | Crown outline | EDJ | Dentinal crown |
| PC1 | 46 | 39 | 25 | 27 | 53 | 45 | 36 | 29 |
| PC2 | 25 | 28 | 13 | 14 | 27 | 31 | 22 | 18 |
| PC3 | 13 | 9 | 10 | 10 | 9 | 8 | 12 | 14 |
| PC4 | 7 | 8 | 9 | 9 | 5 | 5 | 10 | 9 |
| PC5 | 2 | 5 | 7 | 7 | 2 | 4 | 4 | 6 |
| Total % PC1-5 | 93 | 89 | 64 | 67 | 96 | 93 | 84 | 76 |

**Šimková et al. Supporting information**

Shape variation in modern human upper premolars

| **Table B.** **Pairwise comparison of the PERMANOVA analysis results** (*p*-values) between examined geographical populations’ group centroids of all datasets. | | | | | | |
| --- | --- | --- | --- | --- | --- | --- |
| **Dentinal crown** | | | | | | |
| **P3** | | | | | | |
|  | Oceania | South America | Europe | Africa | Southeast Asia | Near East |
| Oceania |  | 0.579 | 0.975 | 0.526 | 0.457 | 0.410 |
| South America | 0.579 |  | 0.709 | 0.823 | 0.183 | 0.153 |
| Europe | 0.975 | 0.709 |  | 0.763 | 0.280 | 0.347 |
| Africa | 0.582 | 0.823 | 0.763 |  | 0.459 | 0.246 |
| Southeast Asia | 0.457 | 0.183 | 0.280 | 0.459 |  | 0.171 |
| Near East | 0.410 | 0.153 | 0.347 | 0.246 | 0.171 |  |
| **P4** | | | | | | |
|  | Oceania | South America | Europe | Africa | Southeast Asia | Near East |
| Oceania |  | 0.840 | 0.807 | 0.454 | 0.433 | 0.728 |
| South America | 0.804 |  | 0.740 | 0.627 | 0.186 | 0.158 |
| Europe | 0.807 | 0.740 |  | 0.572 | 0.509 | 0.715 |
| Africa | 0.454 | 0.627 | 0.572 |  | 0.581 | 0.172 |
| Southeast Asia | 0.433 | 0.186 | 0.509 | 0.581 |  | 0.569 |
| Near East | 0.728 | 0.158 | 0.715 | 0.172 | 0.569 |  |
| **Cervical outline** | | | | | | |
| **P3** | | | | | | |
|  | Oceania | South America | Europe | Africa | Southeast Asia | Near East |
| Oceania |  | 0.6654 | 0.4255 | 0.1553 | 0.6341 | 0.1364 |
| South America | 0.6654 |  | 0.6456 | 0.6966 | 0.6826 | 0.8068 |
| Europe | 0.4255 | 0.6456 |  | 0.3797 | 0.4054 | 0.1728 |
| Africa | 0.1553 | 0.6966 | 0.3797 |  | 0.5637 | **0.0335** |
| Southeast Asia | 0.6341 | 0.6826 | 0.4054 | 0.5637 |  | 0.5687 |
| Near East | 0.1364 | 0.8068 | 0.1728 | **0.0335** | 0.5687 |  |
| **P4** | | | | | | |
|  | Oceania | South America | Europe | Africa | Southeast Asia | Near East |
| Oceania |  | 0.3001 | 0.5617 | 0.2244 | 0.1828 | 0.3915 |
| South America | 0.3001 |  | **0.0014** | 0.1071 | 0.1013 | 0.0555 |
| Europe | 0.5617 | **0.0014** |  | 0.1837 | 0.4417 | 0.1449 |
| Africa | 0.2244 | 0.1071 | 0.1837 |  | 0.2004 | 0.2503 |
| Southeast Asia | 0.1828 | 0.1013 | 0.4417 | 0.2004 |  | 0.4241 |
| Near East | 0.3915 | 0.0555 | 0.1449 | 0.2503 | 0.4241 |  |
| **Crown outline** | | | | | | |
| **P3** | | | | | | |
|  | Oceania | South America | Europe | Africa | Southeast Asia | Near East |
| Oceania |  | **0.0185** | 0.329 | 0.1369 | 0.7294 | 0.4304 |
| South America | **0.0185** |  | 0.302 | 0.6464 | 0.7158 | 0.1842 |
| Europe | 0.329 | 0.302 |  | 0.2094 | 0.6146 | 0.1076 |
| Africa | 0.1369 | 0.6464 | 0.2094 |  | 0.5283 | 0.252 |
| Southeast Asia | 0.7294 | 0.7158 | 0.6146 | 0.5283 |  | 0.3619 |
| Near East | 0.4304 | 0.1842 | 0.1076 | 0.252 | 0.3619 |  |
| **P4** | | | | | | |
|  | Oceania | South America | Europe | Africa | Southeast Asia | Near East |
| Oceania |  | 0.911 | 0.1508 | 0.0963 | 0.1016 | 0.0787 |
| South America | 0.911 |  | 0.2746 | 0.0906 | 0.4864 | 0.3273 |
| Europe | 0.1508 | 0.2746 |  | **0.0068** | 0.1307 | **0.0086** |
| Africa | 0.0963 | 0.0906 | **0.0068** |  | 0.2952 | 0.4801 |
| Southeast Asia | 0.1016 | 0.4864 | 0.1307 | 0.2952 |  | 0.5197 |
| Near East | 0.0787 | 0.3273 | **0.0086** | 0.4801 | 0.5197 |  |
| **EDJ** | | | | | | |
| **P3** | | | | | | |
|  | Oceania | South America | Europe | Africa | Southeast Asia | Near East |
| Oceania |  | 0.1729 | 0.5617 | 0.2295 | 0.5241 | 0.7011 |
| South America | 0.1729 |  | 0.2665 | 0.0706 | 0.1279 | 0.281 |
| Europe | 0.5617 | 0.2665 |  | 0.988 | 0.7473 | 0.9594 |
| Africa | 0.2295 | 0.0706 | 0.988 |  | 0.6718 | 0.6872 |
| Southeast Asia | 0.5241 | 0.1279 | 0.7473 | 0.6718 |  | 0.7868 |
| Near East | 0.7011 | 0.281 | 0.9594 | 0.6872 | 0.7868 |  |
| **P4** | | | | | | |
|  | Oceania | South America | Europe | Africa | Southeast Asia | Near East |
| Oceania |  | 0.8453 | 0.6065 | 0.5863 | 0.4667 | 0.9161 |
| South America | 0.8453 |  | 0.6903 | 0.5115 | 0.7264 | 0.6098 |
| Europe | 0.6065 | 0.6903 |  | 0.7084 | 0.8078 | 0.4025 |
| Africa | 0.5863 | 0.5115 | 0.7084 |  | 0.6685 | 0.732 |

| **Table C. Results of the PERMANOVA analysis.** Differences between group centroids in all datasets (Permutation N: 9999). | | | | | |
| --- | --- | --- | --- | --- | --- |
|  |  | Total sum of squares | Within-group sum of squares | *p*-value | F-value |
| **Dentinal crown** | **P3** | 7574 | 7026 | 0.57 | 0.96 |
|  | **P4** | 8602 | 7985 | 0.60 | 0.95 |
| **Cervical outline** | **P3** | 1987 | 1856 | 0.45 | 1.00 |
|  | **P4** | 2918 | 2663 | 0.06 | 1.32 |
| **Crown outline** | **P3** | 2968 | 2741 | 0.17 | 1.19 |
|  | **P4** | 3350 | 3025 | **0.03** | 1.48 |
| **EDJ** | **P3** | 9213 | 8531 | 0.53 | 0.97 |
|  | **P4** | 1.291E04 | 1.212E04 | 0.87 | 0.82 |

| **Table D. Results of the Mann–Whitney U test**. Differences in dental sizes (expressed by the logarithm of Centroid Sizes) between sexes in upper third and fourth premolars (P3 and P4, respectively). | | |
| --- | --- | --- |
|  | **P3** | **P4** |
| n (F/M) | 9/24 | 9/26 |
| *p*-value | **0.03** | 0.11 |
| Z | 2.14 | 1.56 |

**
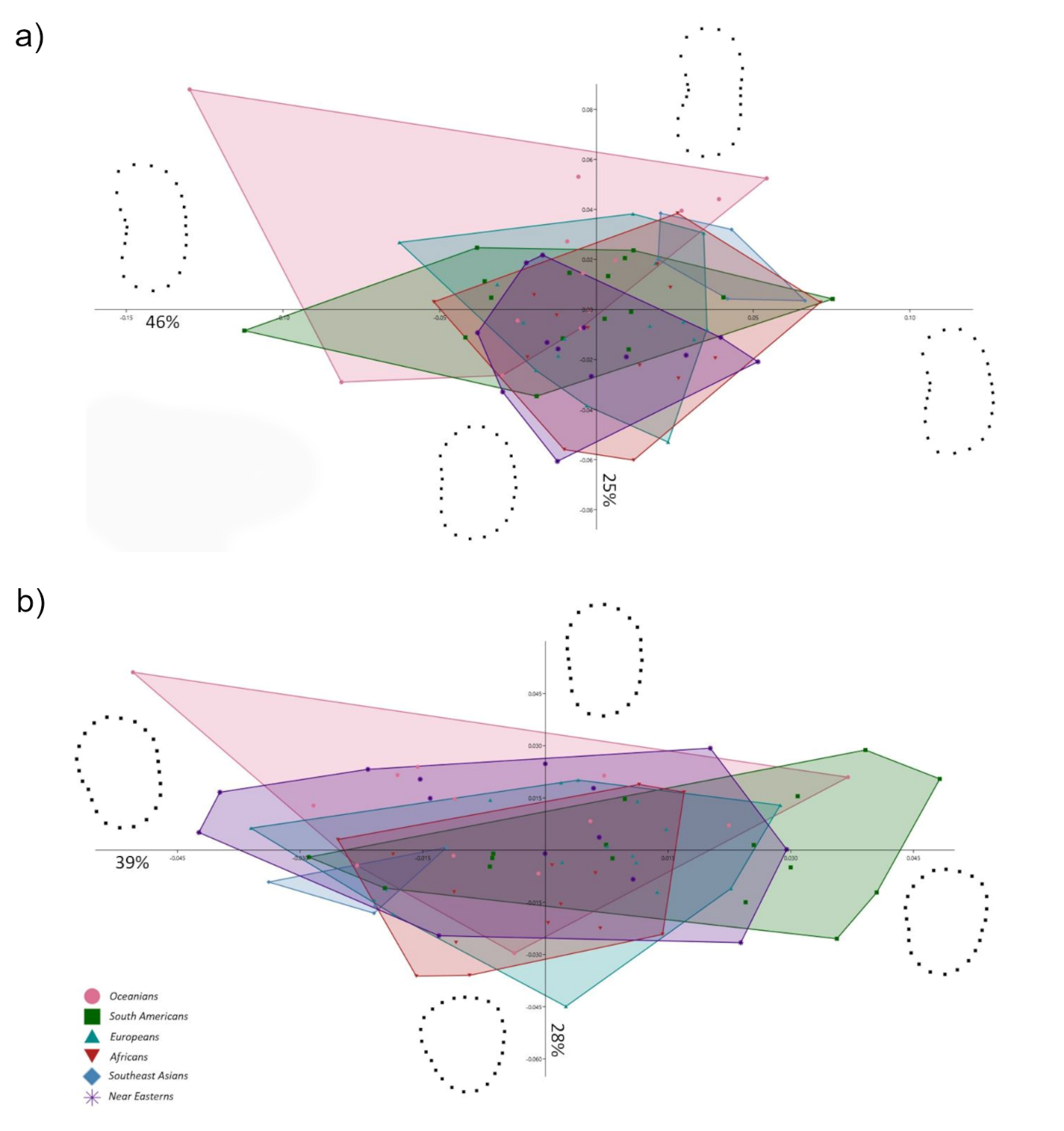
**

Supplementary Figure A. (a) PC1—PC2 plot for third premolars in shape space for the cervical outline (warpings show the real variation in occlusal view at values±0.15/±0.10) and b) the crown outline (warpings show the real variation in occlusal view at values±0.05/±0.06).

Shape variation of the cervical outline along PC1 (46%) was mainly driven by the expansion of the bucco-mesial and mesio-lingual parts of the outline relatively to each other. Along PC2 (25%), the cervical outline varied mainly between round and hourglass-shaped. Oceanians displayed the hourglass-shaped variant more often with a bucco-mesial expansion, while the cervical outlines of African populations were more rounded and mesio-lingually expanded. Shape variation in crown outline along PC1 (39%) reflected the relative expansion of the bucco-distal side, as well as expansion and reduction of the lingual aspect of the tooth either in the mesial or distal direction. Along PC2 (28%) the variation reflected similar findings like in cervical outline, thus the crown outline varied between hourglass-shaped and mesio-distally broad.

**
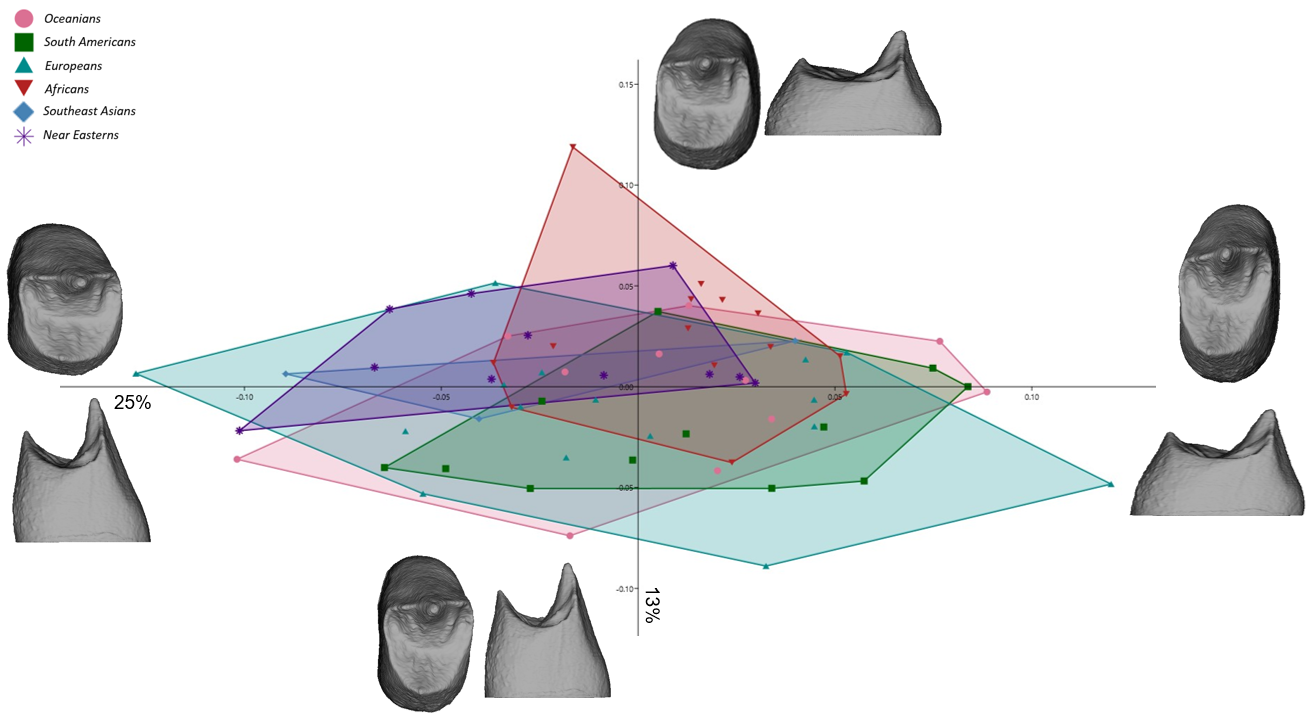
**

Supplementary Figure B. PC1—PC2 plot for third premolars in shape space for the enamel-dentine junction (warpings show the real variation in occlusal view at values±0.15).

The EDJ variation in P3s along PC1 (25%) mostly consists in the relative mesio-distal expansion of the central groove affecting the whole occlusal part of the tooth. The occlusal aspect varied between mesio-distally broad, bucco-lingually constricted (protocone and paracone are shifted towards each other) with wide central groove, and mesio-distally reduced, with main cusps shifted apart from each other due to the mesio-distal reduction of the central groove. Shape variation along PC2 (13%) was driven by the relative expansion of the distal and disto-lingual sides, resulting in either broad and lingually rounded occlusal aspect, or narrow, lingually convergent occlusal aspect.

**
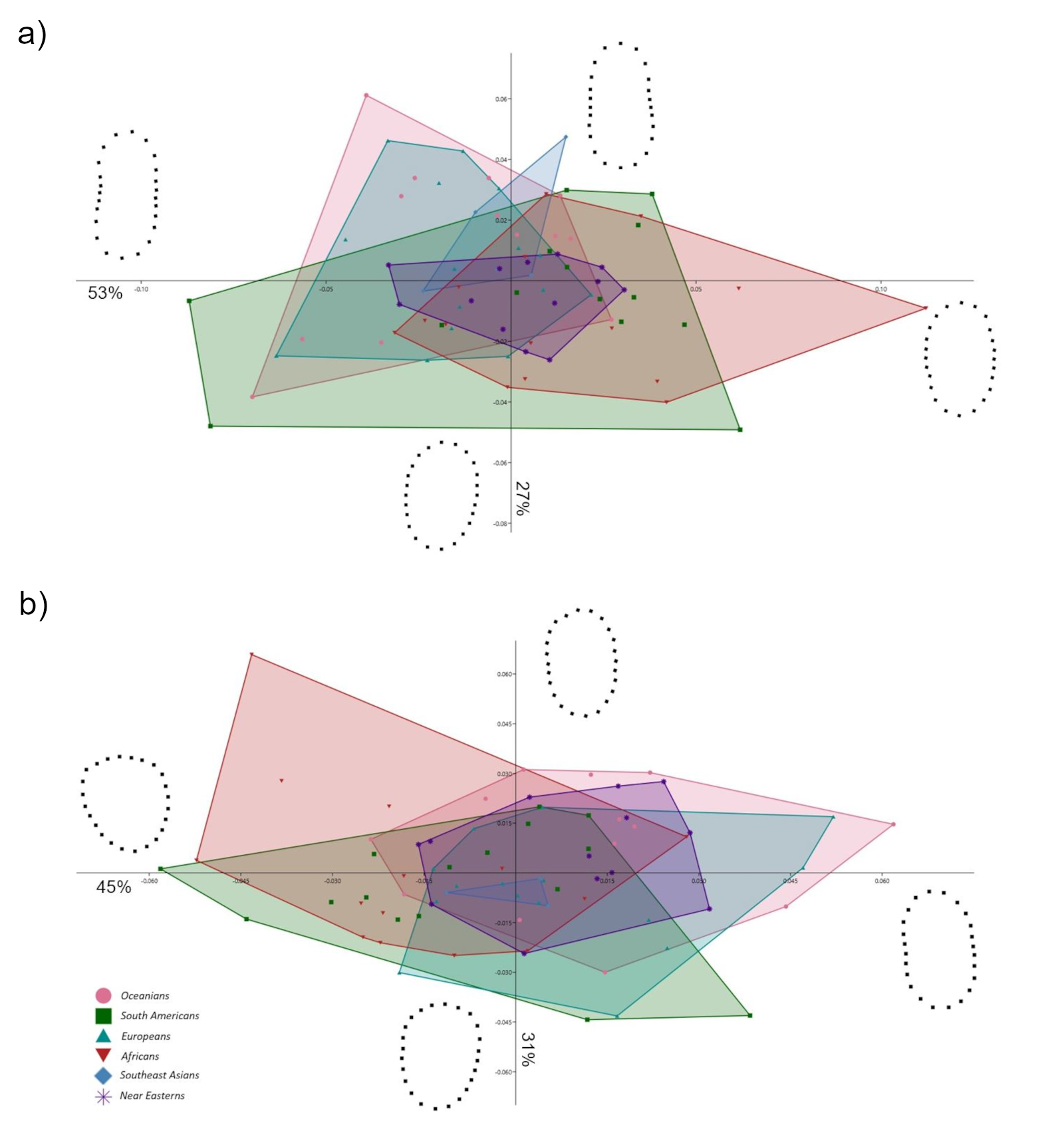
**

Supplementary Figure C. (a) PC1—PC2 plot for fourth premolars in shape space for the cervical outline (warpings show the real variation in occlusal view at values±0.10/±0.08) and b) the crown outline (warpings show the real variation in occlusal view at values±0.07).

For the P4s cervical outline, PC1 (53%) reflected a relative expansion either on the bucco-distal and mesio-lingual, or mesio-buccal and disto-lingual sides of the outline. Variation along PC2 (27%) consisted in the relative mesio-distal constriction in combination with bucco-lingual expansion, resulting in an hourglass shape of the cervical outline.

The shape variation along PC1 (45%) in crown outline reflected the relative mesio-distal constriction and bucco-lingual expansion of the outline, resulting in either hourglass-shaped or rather rounded crown outline. Along PC2 (31%) the shape change reflected relative expansion and reduction of the mesial and distal aspects of the crown outline. Africans show extreme values of the broad, short and bucco-mesially expanded crown outline variation.


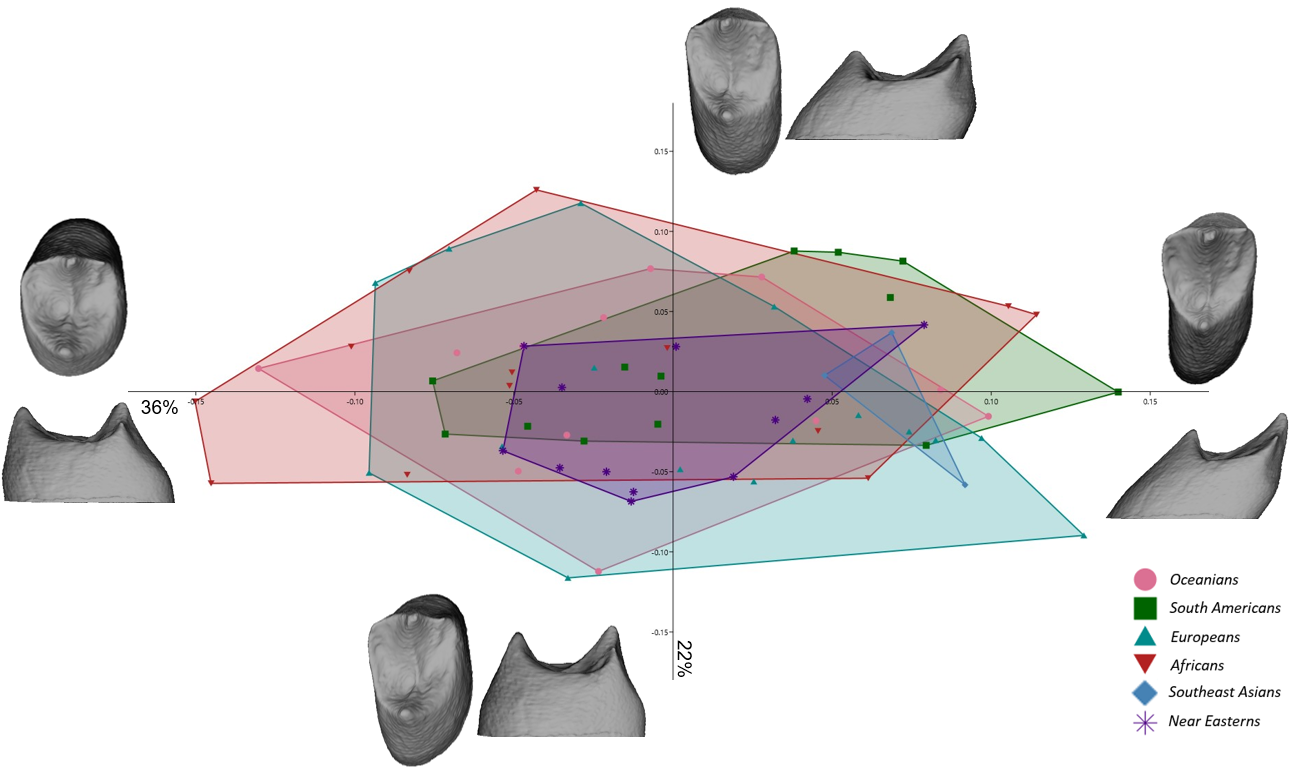


Supplementary Figure D. PC1—PC2 plot for fourth premolars in shape space for the enamel-dentine junction (warpings show the real variation in occlusal view at values±0.15).

The P4s’ EDJ variation along PC1 (36%) reflected mainly two shape types, namely mesio-distally narrow with higher buccal cusp and pointy lingual aspect, or mesio-distally broad, with even cusps’ height and rather rounded lingual aspect. Shape change along PC2 (22%) was driven by the relative position of the lingual cusp shifting either mesially or distally. Mesially positioned buccal cusp was associated with its height increase.

**
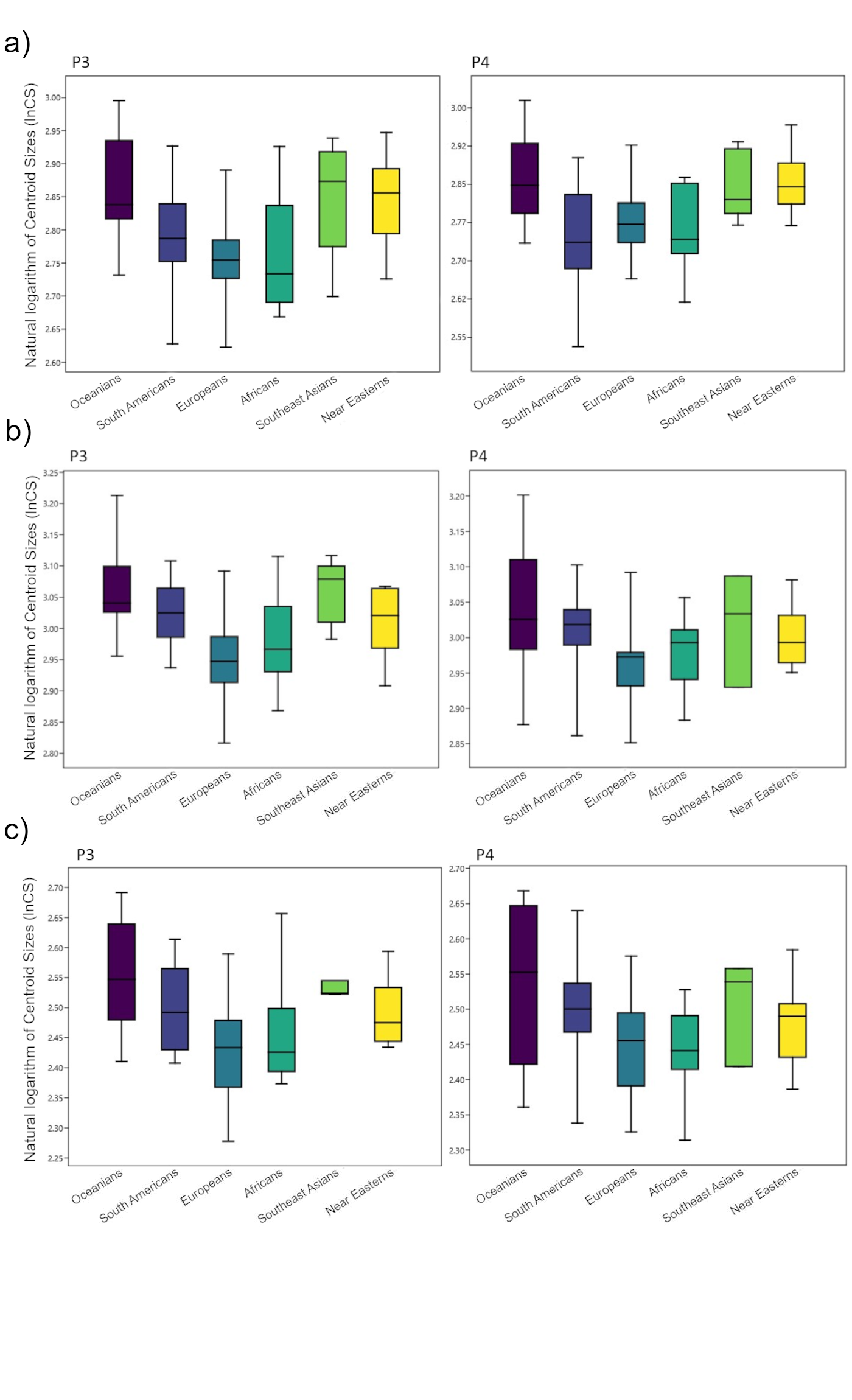
**

Supplementary Figure E. Boxplots of the natural logarithm of Centroid Sizes from the a) cervical outline, b) crown outline and c) the enamel-dentine junction in third and fourth premolars.
